# Intelligent and sustainable waste classification model based on multi-objective beluga whale optimization and deep learning

**DOI:** 10.1007/s11356-024-33233-w

**Published:** 2024-04-18

**Authors:** Gehad Ismail Sayed, Mohamed Abd Elfattah, Ashraf Darwish, Aboul Ella Hassanien

**Affiliations:** 1 School of Computer Science, Canadian International College (CIC), Cairo, Egypt; 2 Misr Higher Institute for Commerce and Computers, Mansoura, Egypt; 3 Faculty of Science, Helwan University, Helwan, Egypt; 4 Faculty of Computers and Artificial Intelligence, Cairo University, Giza, Egypt; 5 College of Business Administration (CBA), Kuwait University, Al Shadadiya, Kuwait; 6 Scientific Research School of Egypt (SRSEG), Cairo, Egypt

**Keywords:** Waste classification, Sustainable waste, TrashNet, Hyperparameter tuning, Beluga whale optimization, Deep learning

## Abstract

Resource recycling is considered necessary for sustainable development, especially in smart cities where increased urbanization and the variety of waste generated require the development of automated waste management models. The development of smart technology offers a possible alternative to traditional waste management techniques that are proving insufficient to reduce the harmful effects of trash on the environment. This paper proposes an intelligent waste classification model to enhance the classification of waste materials, focusing on the critical aspect of waste classification. The proposed model leverages the InceptionV3 deep learning architecture, augmented by multi-objective beluga whale optimization (MBWO) for hyperparameter optimization. In MBWO, sensitivity and specificity evaluation criteria are integrated linearly as the objective function to find the optimal values of the dropout period, learning rate, and batch size. A benchmark dataset, namely TrashNet is adopted to verify the proposed model’s performance. By strategically integrating MBWO, the model achieves a considerable increase in accuracy and efficiency in identifying waste materials, contributing to more effective waste management strategies while encouraging sustainable waste management practices. The proposed intelligent waste classification model outperformed the state-of-the-art models with an accuracy of 97.75%, specificity of 99.55%, F1-score of 97.58%, and sensitivity of 98.88%.

## Introduction

Waste management is at the forefront of many economic and environmental concerns in the current digital era due to the push for full digital transformation. Approximately 1.33 billion out of every 2.01 billion tonnes of municipal solid waste worldwide do not have sustainable management, according to the World Bank (WB) (Bank 2023). With an estimated global trash production of 3.40 billion tons by 2050, daily waste output is expected to increase by 40% or more in low- and middle-income countries (Bank 2023). Only 16% of the world’s population lives in high-income countries, yet they produce 34% of the waste, or 683 million tonnes of waste, according to Bank (2023). Thus, it is more important than ever to solve this issue and to address the growing waste management crisis. The absence of a uniform classification system, which results in inconsistent waste sorting processes, is another key problem (Edjabou et al. 2015). This makes it more difficult to recycle and recover resources effectively. Furthermore, the growth of complicated and composite materials poses a significant obstacle. These materials frequently challenge traditional classification models, necessitating new models to guarantee accurate classification. Additionally, improper classification and separation of hazardous materials pose serious environmental and public health risks (Ahmed et al. 2023a).

To prevent this, effective waste management practices like sorting and recycling are critical for sustainable development. Although manual waste sorting is currently the most accurate approach, it requires trained operators and time-consuming, especially with the exploding amount of waste as urbanization and population growth continue (Abdel-Shafy and Mansour 2018). As a result, automated waste classification models have an immediate need to handle tonnes of waste materials, generating substantial research interest around the world (Chabhadiya et al. 2021). Automated waste classification models can offer a possible solution to optimize waste collection and recycling, resulting in better resource use and lower pollution levels (Kennedy 2018). Additionally, they can have positive effects on the environment as well as the economy by lowering operational expenses (Ruiz et al. 2019). Utilizing these models aligns with the objectives outlined in Target 11 of the Sustainable Development Goals (SDGs), which aim for the development of inclusive, secure, resilient, and sustainable cities and human settlements. Therefore, putting automated waste classification models into place is crucial to accomplishing sustainable development goals in contemporary society, especially in smart cities where the demand for effective waste management models is growing urgently.

Currently, a variety of automated waste sorting methods have been presented, which can be divided into three categories: mechanical, Internet of Things (IoT), and artificial intelligence (AI) (Fu et al. 2021). The mechanical method uses sensors, microprocessors, and mechanical components to automate waste sorting to replace human processes. However, because of its low recognition accuracy, this method frequently fails to achieve proper classification. In contrast, IoT methods aim to address this issue by leveraging cloud servers for increased accuracy, but they are hampered by difficult installation and maintenance procedures, as well as expensive prices. On the other hand, AI methods provide great accuracy, adaptability, and robustness in waste classification. AI-based systems may successfully recognize and classify waste, providing a promising solution to this challenge.

Deep learning is a subset of AI that utilizes neural networks to mimic the human brain’s ability to solve and learn complex problems. Deep learning architectures have impacted a wide range of industries, bringing in revolutionary advances (Balas et al. 2019; Ahmed et al. 2023b). They have gained popularity as a means of extracting high-level features in a variety of domains, including object detection, semantic segmentation, and image classification (Anilkumar and Venugopal 2023). Deep learning improves robustness against noise by automating feature extraction, in contrast to conventional machine learning algorithms. This strategy is a useful tool in a variety of applications since it provides excellent scalability and generalization (Lin et al. 2022b). Additionally, deep learning architectures have proven their efficiency in waste classification, boosting recycling efforts, and contributing to a more sustainable future in the waste management field (Aral et al. 2018; Shi et al. 2021). While deep learning architectures are effective in their ability to model complicated relationships in data, they have significant limitations due to their large number of parameters. The high performance of the deep learning architectures depends on these parameters, which regulate its classification accuracy (Sayed et al. 2021). The model’s tuning and optimization may be complicated by the sheer number of these parameters. improper or inadequate configuration of hyperparameters can lead to problems like underfitting, where the model is too simple to detect underlying patterns in the data, or overfitting, where the model performs exceptionally well on training data but poorly on unseen data. Manually adjusting these hyperparameters can be tedious and time-consuming, requiring substantial knowledge and frequently results in poor settings. Swarm optimization algorithms provide a potential solution to the problem of hyperparameter optimization in deep learning (Bacanin et al. 2023). These algorithms are inspired by the collective behaviors of social creatures such as birds and ants. Through the use of self-organization and collective intelligence, these algorithms present a viable solution by automating the modification of hyperparameters for improved classification model efficiency and accuracy (Sayed 2022; Anitha et al. 2023).

Recent developments in deep learning architectures and optimization-based algorithms have made it possible to accurately detect and classify waste material. Recent models have been proposed for dealing with the waste problem as a classification problem. In Aral et al. (2018), Densenet169, Densenet121, MobileNet, Xception, and InceptionResnetV2 were applied to the waste classification problem. Additionally, Adam and Adadelta were employed as optimizers. Adam’s test accuracy was higher than Adadelta’s. In addition, data augmentation was applied to improve classification accuracy due to limited TrashNet dataset samples. Experiments found the best results with the DenseNet121 using fine-tuning with a 95% accuracy rate. However, the authors’ model obtained a 95% accuracy rate; they employed well-known deep learning architectures without considering hyperparameter optimization of these architectures and handling the class imbalance problem associated with the TrashNet dataset. DenseNet121’s classification accuracy on TrashNet is increased by applying a genetic algorithm (GA) to optimize the fully connected layer of DenseNet121 (Mao et al. 2021). An optimized DenseNet121 was proposed. The proposed optimized DenseNet121 obtained a test accuracy of 99.6%, where 9095 train images and 1013 test images were adopted. However, with these high accuracy results, the authors mentioned that they divided the dataset into 90% training and 10% testing sets. This small testing set could lead to unreliable evaluation metrics and may not accurately reflect the model’s performance on unseen data. Moreover, the authors did not explicitly state whether augmentation was used in the testing set as well, as they only mentioned that the number of images increased from 2527 to 10108. A multi-layer hybrid convolution neural network (MLH-CNN) was proposed by Shi et al. (2021). MLH-CNN was tested and compared to VGG16, AlexNet, and ResNet50 on TrashNet. Accuracy values of 92.6%, 52.2%, 73.1%, and 74.7%, respectively, were obtained. The limitation of MLH-CNN is that the undersampling method is employed to handle class imbalance problems. Reducing the number of instances in the majority class, the undersampling method may lead to loss of valuable information and a less representative sample of the overall population. In Fu et al. (2021), the authors proposed an updated version of MobileNetV3 for automatic garbage classification. The dataset from Huawei’s Garbage Classification Challenge Cup was utilized. In 0.63 s, the proposed garbage classification system is 92.62% accurate. By lowering the number of bottleneck layers and channels, the updated MobileNetV3 may have a lower ability to acquire and express complex features in data. Thus, it may result in low classification performance. In Lin et al. (2022a), RWNet models with various ResNet structures are employed to sort recyclables of the TrashNet dataset. The results revealed that RWNet-101 obtained the highest accuracy with 89.9%. Additionally, the results demonstrated that RWNet models can correctly sort most recyclables except plastic with ROC greater than 0.9. While the authors employed data augmentation techniques and cyclical learning rates to improve the performance of RWNet models, they did not consider the class imbalance problem of the TrashNet dataset. Thus, the maximum accuracy they got was 89.9%. A residual network-based classification model was proposed in Zhang et al. (2021) study. The proposed model was tested on the TrashNet dataset, and a 95.87% accuracy rate was obtained. However, the proposed model obtained a good classification accuracy; deep learning architectures like residual networks have a large number of parameters that need to be optimized to obtain higher classification accuracy.

Most existing models on waste classification focused on employing deep learning architectures or conventional machine learning algorithms, frequently disregarding the essential factor of optimizing their hyperparameter values. Additionally, most of the existing models did not consider the class imbalance problem, and thus, they obtained not high enough waste classification accuracy. As a result, there is a considerable research gap in the development of alternative approaches to improve waste classification models. This paper is driven by the need to fill that gap and further improve the efficiency and accuracy of waste sorting processes in light of recent developments in waste classification models. This paper’s primary goal is to investigate the synergistic potential of the InceptionV3 deep learning architecture with the proposed multi-objective beluga whale optimization (MBWO) for hyperparameter optimization of InceptionV3. By integrating these approaches, the waste classification’s accuracy can be significantly improved, resulting in more effective waste management strategies. Additionally, the class imbalance problem is tackled by employing the random oversampling method followed by data augmentation techniques. The two fundamental phases of the proposed intelligent waste classification model are the classification phase and the data pre-processing phase. The InceptionV3’s hyperparameters are to be optimized during the classification phase using the proposed MBWO-based hyperparameter optimization algorithm.

BWO has proved its efficiency in solving many optimization problems, such as engineering design problems (Jia et al. 2023), feature selection optimization problems (Gao et al. 2023), and simulation optimization problems with stochastic constraints (Horng and Lin 2023). Additionally, it has shown great performance in finding the optimal hyperparameter values of the VGG deep convolutional neural network as discussed in Deepika and Kuchibhotla’s (2024) study, the optimal hyperparameter values of the DeepLabv3-based semantic segmentation architecture as discussed in Anilkumar and Venugopal’s (2023) study, and the optimal hyperparameter values of the convolutional bidirectional long short-term memory with an autoencoder model as discussed in Asiri et al.’s (2024) study. To the best of our knowledge, this is the first time to introduce a multi-objective version of beluga whale optimization for the hyperparameter optimization of InceptionV3 deep learning architecture. Additionally, at the time of writing this paper, a model that is based on a multi-objective version of BWO for hyperparameter optimization of InceptionV3 with the incorporation of data augmentation techniques and random oversampling methods for tackling the class imbalance problem has not been proposed before. The contributions of this paper can be summed up as follows:

- This paper introduces an intelligent waste classification model built upon the proposed optimized InceptionV3 deep learning architecture. This model exhibits the capability to effectively categorize six types of waste, thereby substantially enhancing the accuracy of waste classification.
- A multi-objective version of the beluga whale optimization algorithm is proposed to find the optimal learning rate, dropout period, and batch size hyperparameters of the InceptionV3 deep learning architecture.
- The proposed intelligent waste classification model considers tackling the issue of the imbalanced waste dataset by utilizing data augmentation techniques and the random oversampling method.
- To demonstrate the importance of each component in the proposed model, a thorough evaluation is performed.

This paper is structured as follows: An overview of the materials and methods is carried out in the “Material and methods” section, which provides the foundation for the proposed intelligent waste classification model. In this section, a description of the InceptionV3 architecture and data oversampling techniques are provided. Moreover, the original beluga whale optimization (BWO) algorithm is described. In the “Dataset description” section, the benchmark dataset utilized for the experimentation is described. Then, in the “The proposed intelligent waste classification model” section, the proposed intelligent waste classification model is discussed in detail along with the multi-objective BWO algorithm that is customized for InceptioV3’s hyperparameter optimization. The “Conclusion and future work” section concludes by summarizing the significant findings of this study and providing suggestions for further study endeavors.

## Material and methods

This section provides a summary of two important topics: data oversampling methods for addressing imbalanced datasets and the well-known deep learning architecture InceptionV3. It also explores the fundamental concept and mathematical model of the BWO algorithm, providing insight into the main inspiration and functioning of the algorithm.

### InceptionV3 deep learning architecture

According to Azadi et al. (2023), traditional treatment options have been hampered by rising municipal solid waste generation, waste heterogeneity, and complex waste management and recovery processes. With advancements in computer-based applications, smarter methods for a sustainable environment are required, which are based on recent artificial intelligence (AI) technologies. One of these powerful technologies is deep learning (DL). DL (Menghani 2023) has been widely used in many different fields, including government, science, and business, and has recently emerged as a powerful technique for automatically learning feature representation from data. In the big data era, research on deep learning techniques is becoming popular (Lin et al. 2022a). Through screening the most recent state-of-the-art models in the field of municipal solid waste management (MSWM), most of the models are based on DL architectures (Lin et al. 2022b). Due to their effectiveness, the main motivation of this paper is to introduce a waste classification model based on employing DL architecture (see the “The proposed intelligent waste classification model” section).

InceptionV3 is one of CNN’s deep learning architectures commonly applied for image classification tasks. It has convolution factorization, namely the Inception module. To create deeper networks and handle budget constraints, Inception neural networks were built by lowering dimensionality through the use of 1 × 1 layered convolution. The goal is to acquire numerous kernel sizes inside the network rather than sequentially stacking them and ordering each to function at the same stage. Szegedy et al. (2016) created the first version of the inception architecture in 2012, called GoogLeNet. The suggested model has 27 levels, including inception layers. Convolutional layers 1 × 1, 3 × 3, and 5 × 5 are combined in the inception layer, and their output filter banks are combined into a single output vector that is used as the input to the stage that follows. To further enhance the original model, batch normalization (InceptionV2) and factorization (InceptionV3) were added to reduce computational complexity and parameters while maintaining network performance stability (Szegedy et al. 2015). A convolutional neural network design from the Inception family, InceptionV3, was introduced in 2016 with several improvements, including factorized 7 × 7 convolutions, label smoothing, and the use of an auxiliary classifier to move label information lower down the network.

### Data oversampling

Particularly when dealing with tasks like classification and regression, getting a high-accuracy model from data can be difficult. Skewed class distributions (SCD), which are common in many datasets with uneven class distributions, are a significant barrier. SCD occurs when there are noticeably unbalanced numbers of samples in each class. When resampling data to solve this issue, it is usual practice to undersample the majority and oversample the minority classes (Lin et al. 2023). These approaches aid in balancing the distribution of the classes, enhancing model accuracy, and lowering the danger of bias toward the majority class.

### Beluga whale optimization

Beluga whale optimization, or shortly BWO, is a recent swarm-based metaheuristic algorithm that takes its cues from the collective intelligence of beluga whales. Pair swimming, hunting, and calving are all metaphors for the three phases of discovery, exploitation, and extinction that occur when a whale population collapses. The self-adaptive balance factor and the probability of a whale falling regulate the rate of discovery and extraction in BWO (Zhong et al. 2022).

#### Inspiration

The beluga whale, or Delphinapterus leucas, is a whale shark. The beluga whale is a stocky and stout marine mammal that averages 3.5 to 5.5 m in length and weighs about 1500 kg. Belugas have keen vision and hearing, allowing them to swim and hunt in response to sounds. They also have a cheerful demeanor and graceful movements (Zhong et al. 2022).

#### Mathematical model

The BWO algorithm (Zhong et al. 2022) simulates beluga whale actions such as swimming, hunting, and falling. BWO, like other metaheuristics, consists of an exploration phase and an exploitation phase. By picking beluga whales at random, the exploration step makes sure that the design space can be searched all over the search space. The exploitation phase regulates the designer’s design space’s local search. In swarm optimization algorithms, such as BWO, the used random parameters in the range of 0 to 1 play a critical role in boosting exploration and diversity within the search space. Each parameter’s value is drawn independently using a uniform distribution between 0 and 1, and it has an equal chance of falling anywhere along this range. This randomness introduces a stochastic aspect into the algorithm, preventing it from being stuck in local optima and encouraging exploration of potentially unexplored areas of the solution space. Furthermore, stochasticity benefits in preventing premature convergence to undesirable solutions, which might occur if the algorithm follows a deterministic route. Therefore, the inclusion of random parameters encourages a balanced exploration–exploitation strategy, enabling the swarm algorithm to traverse the solution space rapidly and converge on globally optimal solutions.

##### BWO’s exploration

1 $${B}_{f}={B}_{0}(1-\frac{T}{2{T}_{{\text{Max}}}})$$

where $T$ is the current iteration, ${T}_{{\text{Max}}}$ is the largest possible iteration, and ${B}_{0}$ is a column vector of random numbers with dimensions 30 × 1. Each element of the ${B}_{0}$ vector is generated at random from a uniform distribution between [0, 1]. When ${B}_{f}$ is greater than 0.5, exploration is occurring, and when it is less than 0.5, exploitation is occurring.

At this phase, the beluga whale pair swim is what determines the positions of search agents, and the positions of beluga whales are updated as follows:

2 $${X}_{i,j}^{T+1}=\left\{\begin{array}{ll}{X}_{i,j}^{T}+({X}_{i,j}^{T}-{X}_{i,pj}^{T})(1+r1){\text{sin}}(2\pi r2)& j=\mathrm{even\;numbers}\\ {X}_{i,j}^{T}+({X}_{i,j}^{T}-{X}_{i,pj}^{T})(1+r1){\text{cos}}(2\pi r2)& j=\mathrm{odd\;numbers}\end{array}\right\}$$

where $pj (j=\mathrm{1,2},...,d)$ is a random number chosen from the d-dimension, $T$ is the current iteration, ${X}_{i,j}^{T+1}$ the new location for the *i*th beluga whale on the *j*th dimension, and to improve the random operators in the exploration phase, two random numbers, $r1$ and $r2$, are employed.

##### BWO’s exploitation

3 $${X}_{i,j}^{T+1}=r3\times {X}_{{\text{best}}}^{T}-r4{\times X}_{i,j}^{T}+C1\times {L}_{f}({X}_{r,j}^{T}-{X}_{i,j}^{T})$$

where ${X}_{{\text{best}}}^{T}$ is the best position among beluga whales, ${X}_{i,j}^{T}$ is the current positions for the *i*th beluga whale, ${X}_{r,j}^{T}$ is the *r*th random beluga whale, ${X}_{i,j}^{T+1}$ is the *i*th new position of beluga whale, $r3$ and $r4$ are random values falling between [0, 1], and $C1=2r4(1-\frac{T}{{T}_{{\text{Max}}}})$ is the strength of the random jump.

4 $${L}_{f}=0.05\times \frac{u\times \sigma }{{|v|}^{1/\beta }}$$

where ${L}_{f}$ is the Levy flight parameter. It is used in the position’s updating of BWO during the exploitation phase. $u$ and $v$ are random values with normal distributions.

5 $$\sigma =\left(\frac{\tau (1+\beta )\times {\text{sin}}(\pi \beta /2)}{\tau ((1+\beta )/2)\times \beta \times {2}^{(\beta -1)/2}}\right)$$

where $\beta$ is a constant number equal to 1.5, and $\pi$ is a constant number equals to 3.14. $\tau$ is a function that extends the factorial function to a non-integer of its input value.

##### BWO’s fall

The beluga whales face danger from polar bears, killer whales, and people throughout their migration and foraging. Because they are generally intelligent animals, belugas can avoid danger by exchanging information with one another. Many other creatures are fed as a result of the phenomenon known as whale fall (Zhong et al. 2022). It is possible to express the mathematical model as defined as follows.

6 $${X}_{i,j}^{T+1}=r5{X}_{i,j}^{T}-r6{X}_{r,j}^{T}+r7{X}_{{\text{step}}}^{T}$$

where $r5$, $r6$, and $r7$ are random numbers between [0, 1], and ${X}_{{\text{step}}}^{T}$ is the step size of whale fall created as follows:

7 $${X}_{{\text{step}}}^{T}=({u}_{b}-{l}_{b}){\text{exp}}(-C2T/{T}_{{\text{Max}}})$$

where ${u}_{b}$ and ${l}_{b}$ are the upper and lower bounds of the variables, and $C2$ is the step factor linked to the likelihood of whale fall and population number $(C2=2{W}_{f}\times n)$. The design parameters, the number of iterations, and the maximum number of iterations all affect the step size. In BWO, the probability of whale fall ${W}_{f}$ is computed as a linear function and defined as follows.

8 $${W}_{f}=0.1-0.05T/{T}_{{\text{Max}}}$$

The probability of a whale decreasing from 0.1 in the first iteration to 0.05 in the final iteration demonstrates that the risk to beluga whales decreases as they approach their food source during the optimization process.

## Dataset description

**Fig. 1:**
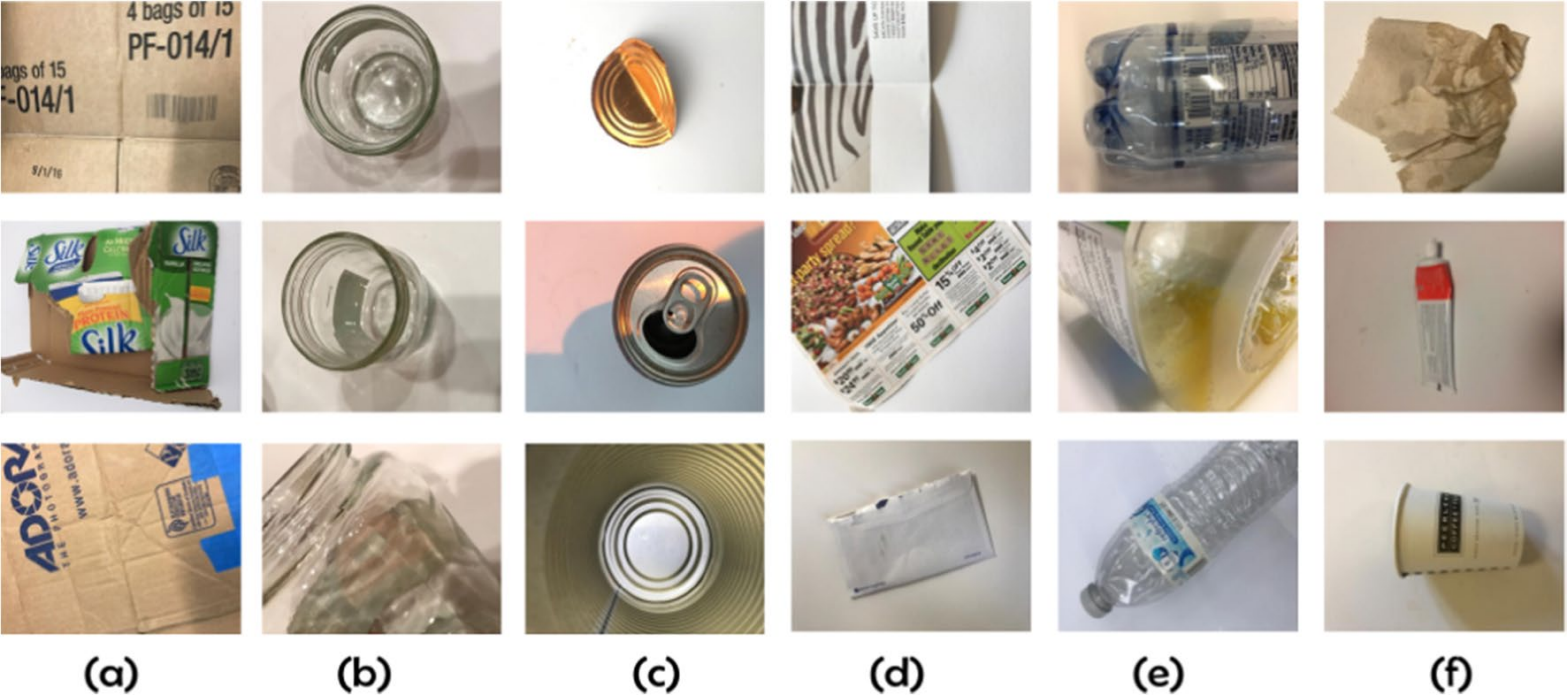
Sample of TrashNet dataset: **a** cardboard, **b** glass, **c** metal, **d** paper, **e** plastic, and **f** trash

A benchmark dataset, namely TrashNet, is adopted for evaluation of the proposed model’s performance (Yang and Thung 2016). The 2573 images in the adopted dataset each show a single product made of one of the six different classes of materials: cardboard, glass, metal, paper, plastic, and garbage. All of the images have a white or cardboard background with a resolution of 512 × 384 pixels. The cell phone camera was used to record the dataset, which was taken both inside and outside. It has since been available to the general public for research. Figure 1 shows samples of the adopted TrashNet dataset.

**Fig. 2:**
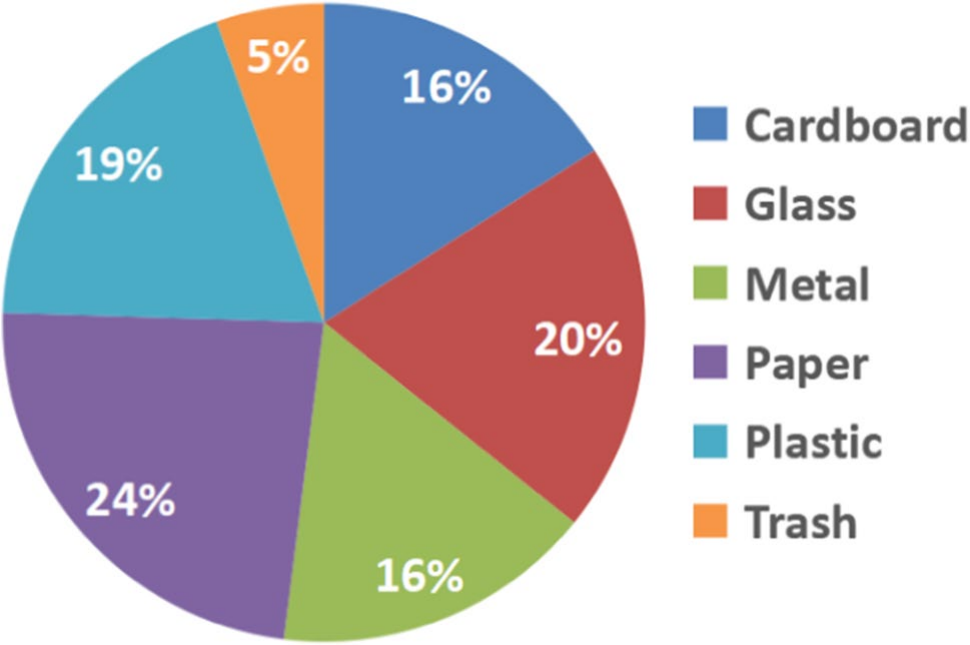
The class distribution of TrashNet dataset

The adopted dataset’s class distribution suffers from a class imbalance problem as can be observed from Fig. 2. The trash class is a minority class since it has a lot fewer samples than the other classes. Class imbalance can have a negative influence on machine learning model performance since the model may be biased toward the majority class. Therefore, to ensure a fair assessment of the performance of the waste classification model, this paper employs proper approaches to address the class imbalance problem.

## The proposed intelligent waste classification model

**Fig. 3:**
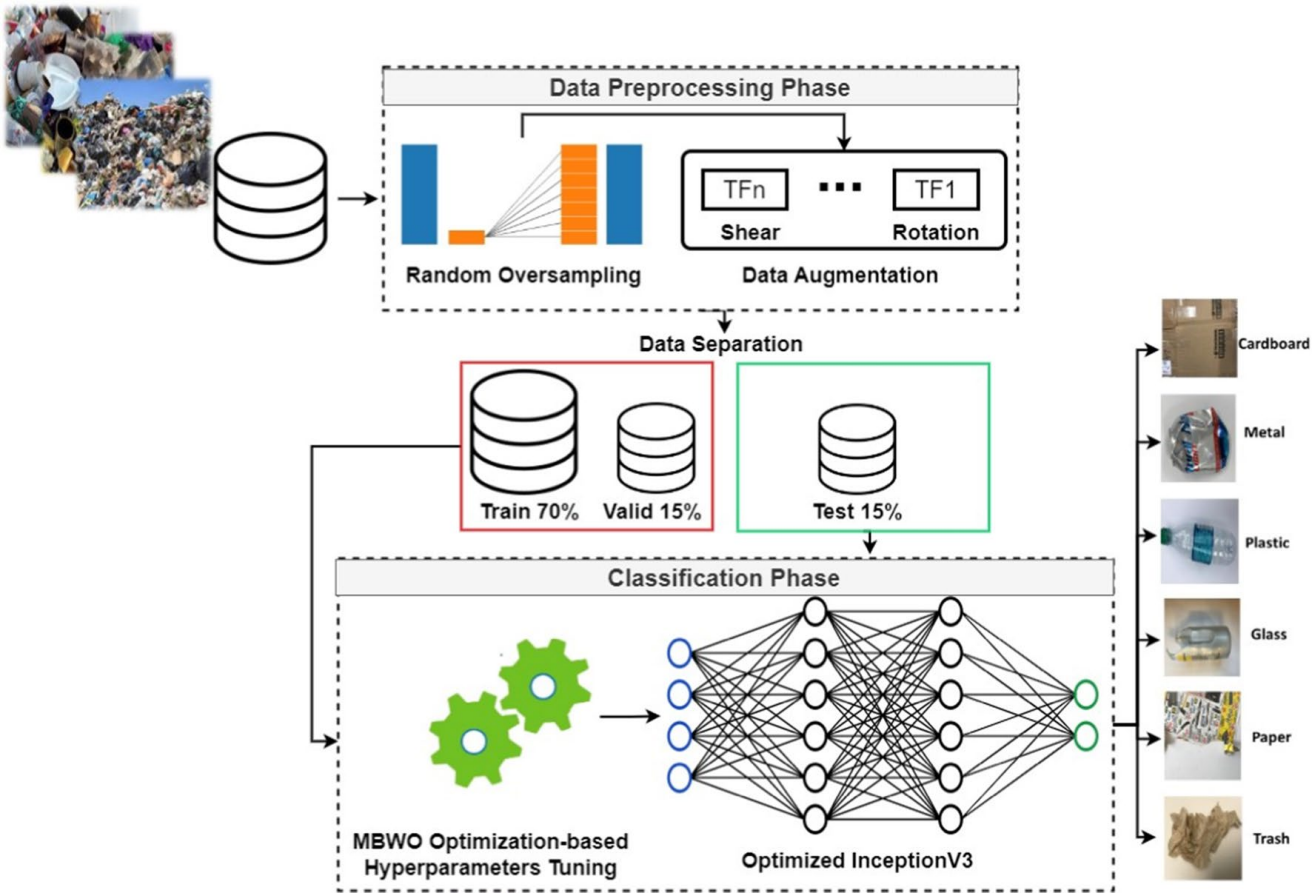
The general architecture of the proposed intelligent waste classification model

A new model for automatic waste classification is presented in this paper. The optimized InceptionV3 deep learning architecture serves as the primary foundation for the proposed model. Data pre-processing and classification based on using the proposed multi-objective beluga whale optimization-based hyperparameter tuning of InceptionV3 comprise its two primary phases. Figure 3 shows the overall architecture of the proposed intelligent waste classification model. In the preprocessing phase, three main methods are applied. These methods are image resizing followed by data oversampling and data augmentation. First, the original images are resized to 299 $\times$ 299 $\times$ 3. Then, the random oversampling method is applied to tackle the class imbalance problem. Then, to reduce the overfitting issue and improve the InceptionV3 deep learning architecture’s generalization capability, data augmentation techniques are applied. Then, the processed dataset is divided into 70% for training, 15% for testing, and 15% for validation. The training and validation sets are used for hyperparameter optimization of InceptionV3, while the testing set is used to evaluate the performance of the overall proposed intelligent waste classification model. Finally, in the classification phase, a multi-objective variant of beluga whale optimization (MBWO) is proposed. The proposed MBWO-based hyperparameter tuning algorithm is applied to find the best hyperparameter settings for the InceptionV3 deep learning architecture. Then, the optimized InceptionV3 is utilized to classify each test image of the adopted TrashNet dataset.

It is crucial to explain how the model interacts with current systems to guarantee seamless integration with current waste classification systems. The proposed intelligent waste classification model functions as an adjunct infrastructure module that smoothly interfaces with the current waste classification system. This integration happens after the post-segregation stage when waste materials are classified. This integration stands out due to its remarkable autonomy. The proposed model runs without the need for human intervention, demonstrating a useful and economical improvement to current waste management practices. By automating the waste classification process, efficiency and accuracy are significantly improved. This approach enhances the accuracy of waste classification while ensuring harmonious compatibility with current waste classification systems by utilizing the complimentary characteristics of both existing technology and the proposed optimized InceptionV3 deep learning architecture. Next, a detailed description of each phase in the proposed model is presented in the upcoming subsections.

### Data pre-processing phase

During this phase, the annotated images are scaled to 299 $\times$ 299 $\times$ 3 after detecting each object. This step is essential to guarantee that the resized images can fit into the input layer of the InceptionV3 deep learning architecture. Two common techniques—data oversampling and data augmentation—are applied to address the problem of class imbalance. The accuracy and robustness of the proposed model are significantly increased by using these strategies.

#### Data oversampling

This paper uses the TrashNet dataset, which contains 2573 images of six different classes of materials, including cardboard, glass, metal, paper, plastic, and trash. With some classes having fewer samples than others, each class’s sample count varies. The adopted dataset has 403 cardboard, 501 glass, 410 metal, 594 paper, 482 plastic, and 137 trash images. As can be observed, just 5% of the total is made up of the rubbish class, with the remaining classes making up the remaining 16 to 24%. This paper addresses the issue of class imbalance in the adopted dataset to provide a fair and accurate classification of all classes.

Most machine learning algorithms with deep learning architectures suffer from this type of dataset. This is because the majority of the information is connected to the dominant category, which might lead to the misclassification of other smaller categories. One method for dealing with class imbalance is to randomly resample the dataset. To randomly resample an unbalanced dataset, there are two well-known methods, the first one known as undersampling and the second one as oversampling. In the undersampling method, the instances from the majority class are discarded, while, in the oversampling method, the instances are duplicated in the minority class. In this study, the minor categories’ size is increased using the random oversampling approach (ROS), which involves randomly replicating some of the images. This method ensures that each class’s distribution has the same size as the distributions of the other classes. As a result, it can impressively prevent overfitting CNN architecture.

#### Data augmentation

Several data augmentation techniques were applied in this paper to improve the InceptionV3 deep learning architecture’s generalization capability and reduce the overfitting issue. In this paper, random geometric transformations including random translation, random flipping, random scaling, random shearing, and random rotation in the *X*- and *Y*-axes are presented. By utilizing these techniques, the model becomes more resilient to changes in the input data, resulting in enhanced functionality and accuracy. The used data augmentation techniques are random *Y* and *X* reflections, random *Y* and *X* translations with random values in range [− 30, + 30], random rotation angles with random angle values in range [− 3, + 3], random *X* and *Y* shears with random factor in range [− 0.05, + 0.05], and random *X* and *Y* scales with random factor in range [0.8, + 1.2].

### Classification phase

This stage involves feeding the optimized version of InceptionV3—which is based on the proposed multi-objective beluga whale optimization (MBWO)—with the processed images dataset. The dataset is divided into three sets, train, test, and valid sets. The train and valid sets are used to feed the proposed MBWO-based hyperparameter optimization algorithm. Then, the optimal hyperparameter values are reported. Finally, the test set is used to evaluate the performance of the optimized InceptionV3 deep learning architecture. Next is the detailed description of each subphase.

#### An improved beluga whale optimization: multi-objective version for InceptionV3 hypermedia optimization

Although deep learning algorithms have achieved unprecedented success in several different applications, the accuracy of these algorithms mainly depends on their hyperparameters. The selection of the values of these hyperparameters is often done by an expert. Thus, optimizing these parameters is considered a substantial obstacle to developing a deep learning architecture. This work introduces a multi-objective version of the BWO algorithm to find the optimal hyperparameter values of the InceptionV3 deep learning architecture. The proposed multi-objective BWO, or shortly, MBWO-based hyperparameter tuning algorithm iteratively finds the best values of the learning rate, dropout period, and batch size hyperparameters. Next is a detailed description of the proposed MBWO-based hyperparameter tuning of the InceptionV3 algorithm.

##### Parameter initialization

The proposed algorithm begins by setting the maximum number of iterations to 20, the dimensionality size to 3, the population size to 30, and the number of epochs to 15. Each position of a beluga whale consists of values of ${Y}_{1}$, ${Y}_{2}$, and ${Y}_{3}$, where ${Y}_{1}$ represents the learning rate, ${Y}_{2}$ represents the batch size, and ${Y}_{3}$ represents the dropout period. The bounding value range for ${Y}_{1}$ is set between [0.00001, 0.0005]; for ${Y}_{2}$, it is set between [1, 128]; and for ${Y}_{3}$, it is set between [1, 10].

##### Fitness function

The MBWO algorithm experiments with different combinations of learning rate, dropout period, and batch size during each iteration of the optimization process. By training the InceptionV3 deep learning architecture on a portion of the available data, it assesses its performance for each combination. The MBWO algorithm determines whether to increase or reduce the values of the hyperparameters by evaluating the model’s performance after each iteration. This is done by calculating the fitness function per each beluga whale’s position.

To get the optimum InceptionV3 hyperparameter values that maximize both specificity and sensitivity, this paper offers an optimization approach designed for tackling multi-objective issues. Pareto fronts have historically been a popular method in multi-objective optimization. However, creating Pareto fronts can be a resource and computationally intensive (Nalluri et al. 2017). To overcome this challenge, this paper proposed a different strategy that includes linearly combining multi-objective functions to create a single linear compound objective function. The optimization procedure is shortened while still producing the required results by integrating several objective functions into a single unified function. The multi-objective functions are linearly integrated, with weights given to each function, to produce this single objective function. The proportional importance or priority assigned to each target is determined by these weights. The optimization process can be directed to develop solutions that strike the ideal balance between specificity and sensitivity by carefully choosing and allocating these weights. In comparison to conventional methods depending on Pareto fronts, the optimization process is more effective and computationally tractable when utilizing this linear combination approach. It enables a more focused exploration of the search space, which results in the discovery of classifier parameter values that provide the optimal specificity and sensitivity trade-off.

Sensitivity, commonly referred to as the true positive rate, measures how well a classifier can recognize positive samples. It quantifies the proportion of actual positive samples that are correctly classified as positive. When minimizing false negatives or making sure that positive samples are not incorrectly classified as negative, sensitivity must be maximized. However, specificity, also known as a true negative rate, assesses the classifier’s accuracy in identifying negative cases. It displays the percentage of samples that are appropriately labeled as negative but are truly negative. When reducing false positives or making sure that negative samples are not mistakenly classified as positive, maximizing specificity is crucial. The proposed MBWO algorithm seeks to balance these two performance indicators by considering both sensitivity and specificity as optimization targets. Because they focus on the right classification of minority and majority classes, sensitivity and specificity are well-suited for imbalanced data as will be further discussed in the upcoming sections. While boosting specificity enables precise classification of negative samples, achieving high sensitivity ensures that positive instances are properly identified. To evaluate how good the position of a beluga whale is, let the classifier being created to categorize a given dataset be a binary class. Thus, sensitivity and specificity are applied to determine the classifiers’ performance parameters. Equations (9) and (10) show the mathematical formula of specificity (*SP*) and sensitivity (*SN*). The mathematical definition of the overall adopted fitness function is shown in Eq. (11).

9 $$SP=\frac{TN}{TN+FP}$$

10 $$SN=\frac{TP}{FN+FP}$$

where *FP* stands for false positive samples, *FN* for false negative samples, *TN* for true negative samples, and *TP* for true positive samples.

11 $$\mathrm{Maximize\;}{F}^{*}={W}_{1}\times SP+{W}_{2}\times SN,\mathrm{ where\;}{W}_{1}+{W}_{2}=1$$

where ${W}_{1}$ and ${W}_{2}$ are constant parameters and each parameter equals 0.5.

##### Beluga’s position updating

The proposed MBWO continuously reduces the search space and improves the values of the learning rate, dropout period, and batch size through this iterative process. It searches for the set of hyperparameter values that maximizes the model’s efficiency. The positions of the beluga whales are analyzed according to their fitness values throughout each iteration of the algorithm, and the position with the highest fitness value is regarded as the optimal one. Through the optimization process, each beluga’s position is updated according to Eqs. (2) and (3).

##### Termination criteria

**Fig. 4:**
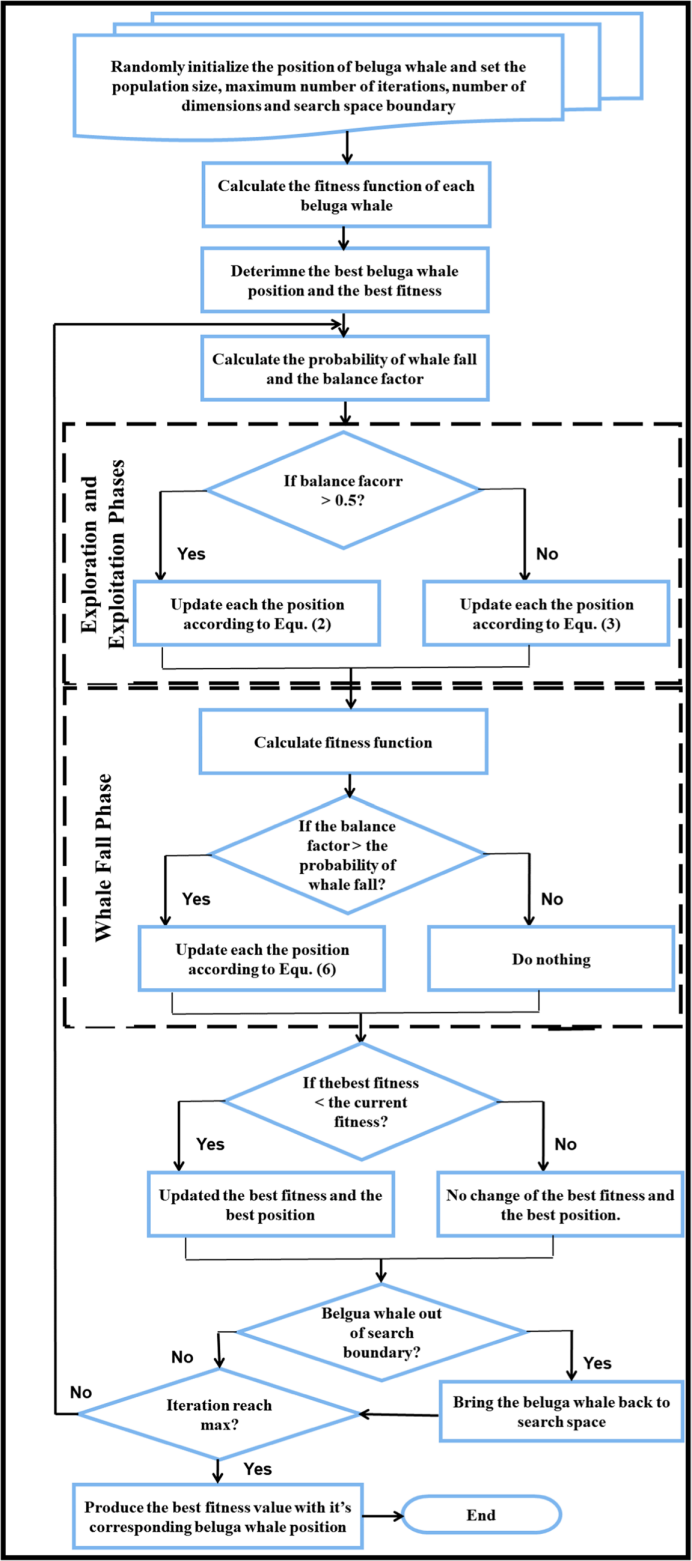
The flowchart of the proposed MBWO-based hyperparameter optimization of InceptionV3 algorithm

When the optimization process should end is decided by the termination criterion. The maximum number of iterations is used in this paper to define the termination criterion. Once this requirement is met, the MBWO algorithm reports the best beluga whale position, which is the optimal combination of dropout period, learning rate, and batch size that produced the best performance during the optimization process. The overall flowchart of the proposed MBWO-based hyperparameter optimization algorithm is shown in Fig. 4.

#### Optimized InceptionV3

In this step, the MBWO-based hyperparameter optimization algorithm is used to meticulously search for the optimal values of the hyperparameters within the InceptionV3 algorithm. The dropout period, learning rate, and batch size are the focus of the proposed optimization algorithm, which is critical for improving the performance of the InceptionV3 deep learning model. The model is prepared for classification tasks after the process of hyperparameter optimization is complete. With high accuracy and effectiveness, the InceptionV3 deep learning architecture excels at classifying objects within images.

When classifying an image, the model runs the input image through several convolutional layers to extract complex features and patterns. Fully connected layers then examine and interpret these retrieved features, enabling the model to comprehend the intricate relationships present in the image. The dropout period, learning rate, and batch size optimized hyperparameter values all contribute to the model’s capacity to generalize and generate precise predictions.

By utilizing the optimized hyperparameter values obtained through the MBWO-based optimization, the InceptionV3 model is properly tuned and optimized to recognize a variety of objects and accurately classify them. It can effectively distinguish between multiple classes and produce accurate classification results due to its deep architecture design and effective parameter settings, as will be shown in the result section.

## Experimental results and discussion

This section assesses and compares the performance of the proposed intelligent waste classification model using the multi-objective beluga whale optimization and InceptionV3 deep learning architecture to the state-of-the-art models. Several measurements are adopted. These measurements are accuracy, specificity, sensitivity, F1-score, receiver operating characteristic (ROC) curve, and convergence curve. These measurements provide information about several elements of the model’s performance. Specificity assesses the model’s ability to correctly identify instances that do not belong to a certain class. It represents the proportion of true negative instances to the overall number of negative instances. Specificity in waste classification helps measure how well the model avoids misclassifying occurrences as one sort of waste material when they belong to another. High specificity results in fewer false positives. Accuracy assesses the overall correctness of the model’s predictions. It is the proportion of correctly classified instances to the total number of instances. In a waste classification model, accuracy refers to the model’s ability to correctly classify images across all waste material classes. However, accuracy alone may not be sufficient if the classes are uneven or if some sorts of misclassifications are more significant than others. Sensitivity assesses the model’s ability to correctly identify instances of a given class. It is the ratio of true positive instances to the total number of positive instances. In a waste classification model, sensitivity is used to examine how successfully the model detects each category of waste material. A high sensitivity indicates that the model will miss fewer instances of a specific waste material. The F1-score is the harmonic average of precision and recall (sensitivity). It strikes a balance between precision and recall, making it a valuable metric in situations with an unequal class distribution. It is especially useful in waste material classification since it provides an overall evaluation of the model’s performance, taking into account both the ability to accurately identify occurrences of each class and the ability to avoid misclassification.

Six primary experiments are carried out. The first experiment aims to analyze the adopted dataset. Additionally, in this experiment, the proposed model’s performance is compared before and after applying each component of the proposed model to show the significance of each component in the classification result. Additionally, different oversampling methods are compared. The performance of the proposed MBWO-based hyperparameter optimization is compared with other swarm intelligence algorithms. In this experiment, the mean, and the standard deviation of the obtained fitness value are utilized. Additionally, the *P-*value from Wilcoxon’s rank is calculated. Furthermore, the classification results are compared following the determination of optimal hyperparameters of the adopted swarm-based hyperparameter optimization algorithms. The third experiment aims to show the importance of finding the optimal hyperparameter values of InceptionV3. In this experiment, the effects of the batch size, the learning rate, and the dropout period are analyzed. Additionally, several statistical tests are utilized to analyze these parameters and show whether they are statistically significant and can affect the performance of InceptionV3. The fourth experiment aims to evaluate the overall performance of the proposed intelligent waste classification model. In this experiment, the ROC, confusion matrix figure, and training progress curve are utilized. The fifth experiment aims to compare the performance of the proposed optimized InceptionV3 with other well-known deep learning architectures. Finally, in the last experiment, the performance of the proposed intelligent waste classification model was compared with the state-of-the-art models.

In all the conducted experiments, the best results are highlighted to make it easier for the reader to keep track of the best-obtained results in each experiment. Additionally, all the conducted experiments are implemented and tested on MATLAB 2020 with 16 GB RAM and Core i7. The deep learning toolbox was employed to facilitate the implementation of the proposed model.

**Fig. 5:**
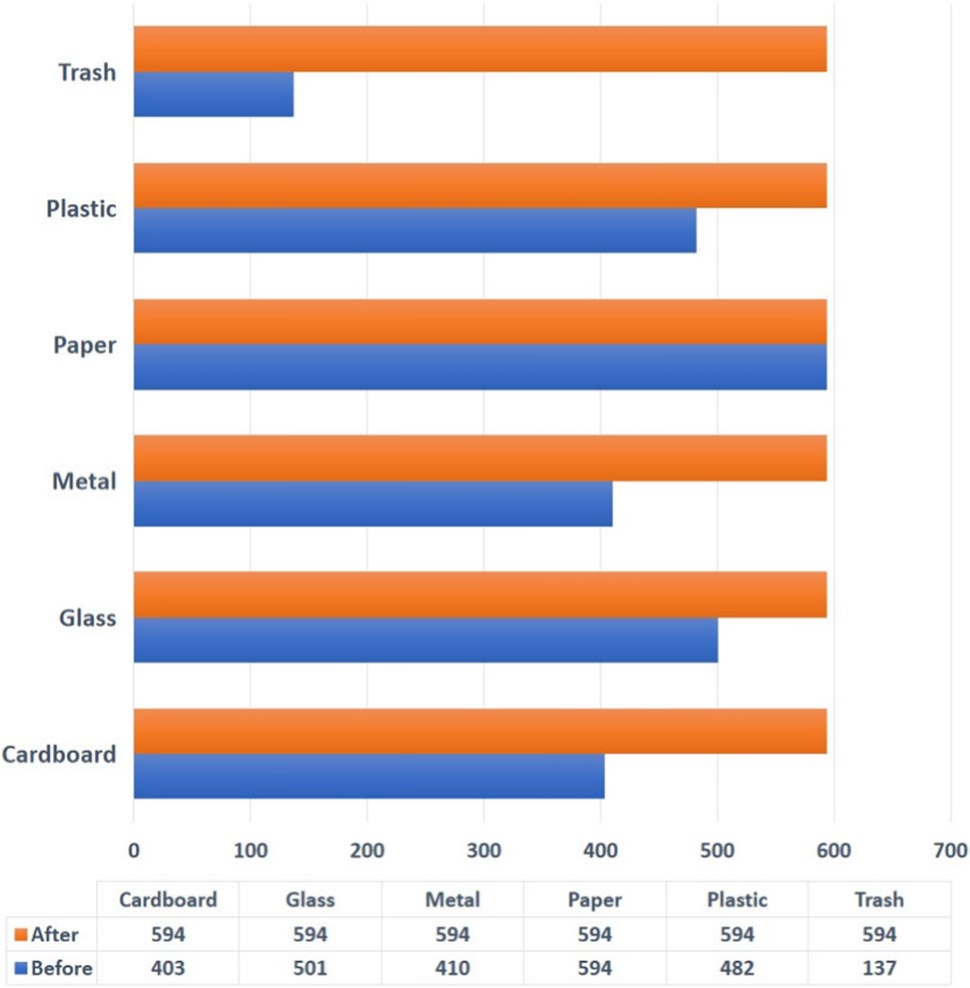
The class distribution before and after applying random oversampling method

In the first experiment shown in Fig. 5, the class distribution of the dataset before and after the random oversampling method was employed. In comparison to other classes like paper, plastic, cardboard, glass, and metal, the figure shows that the number of samples for the trash class is significantly fewer. The model’s ability to accurately recognize trash may suffer from this class imbalance problem. The random oversampling method is therefore needed to address this problem and guarantee a balanced dataset by creating artificial samples for the minority class.

The purpose of the experiment in Table 1 is to show that each component of the proposed model makes a significant contribution to the overall effectiveness of the proposed intelligent waste classification model. In this table, a comparison of the waste classification model’s performance before and after applying each component of the proposed model including the random oversampling technique, random oversampling (ROS), and the proposed MBWO-based hyperparameters optimization of InceptionV3 is shown. The employed TrashNet dataset has a problem with imbalance, with the trash class, a minor class, having a much smaller number of samples than the other major classes. The classifier’s performance may be adversely affected by this problem. Before using the random oversampling method, classification accuracy, F1-score, sensitivity, and specificity were poor. This indicates that the trash class was incorrectly classified by the classifier. In addition, the classification of the major classes was skewed because of the large disparity in sample counts between the trash class and other important classes, such as the paper class. As a result, the classification model’s accuracy, F1-score, sensitivity, and specificity were decreased. The classifier’s performance greatly increased after using the random oversampling method, demonstrating how well this method handles the issue of class imbalance. Also, Table 1 compares the performance of the proposed waste classification model before and after using data augmentation. In this experiment, only the data augmentation phase is removed from the proposed model, while the other phases are left. According to the findings in Table 1, the issue of overfitting in a CNN architecture can be successfully mitigated by applying a variety of data augmentation techniques, which enhances the overall model’s performance. This table also includes a comparison of the performance of the proposed waste classification model before and after utilizing the proposed MBO-based hyperparameter selection approach. In this paper, three hyperparameters that are crucial for optimizing deep learning models were used: learning rate, dropout period, and batch size. The initial model evaluation employed the default values for these hyperparameters. The outcomes show that choosing the ideal settings for these hyperparameters can considerably affect the accuracy, F1-score, sensitivity, and specificity performance of the InceptionV3 CNN architecture. The table sheds light on the efficiency of the MBWO algorithm in choosing the appropriate hyperparameters, enhancing the overall performance of the proposed waste classification model. Also, Table 1 compares two oversampling methods for the proposed waste classification model: random oversampling (ROS) and synthetic minority oversampling method (SMOTE). SMOTE is a well-known oversampling technique that produces synthetic samples in the minority classes by locating the minority data points’ k-nearest neighbors and basing new samples on the information from these neighbors (Barua et al. 2011). However, in this paper, the experimental outcomes showed that ROS is the best oversampling method for addressing the problem of class imbalance in the adopted dataset. Therefore, in the proposed intelligent waste classification model, ROS was employed to oversample the minority class samples.

**Table 1:** The performance of the proposed intelligent waste classification model before and after applying each component of the proposed model

	Oversampling			Augmentation		MBWO	
	Before	After (ROS)	After (SMOTE)	Before	After	Before	After
Specificity (%)	99.06	99.55	97.42	97.55	99.55	95.78	99.55
Sensitivity (%)	93.33	98.88	90.21	90.13	98.88	89.26	98.88
Accuracy (%)	85.98	97.57	94.57	93.32	97.57	90.76	97.57
F1-score (%)	84.15	97.58	94.72	93.39	97.58	90.89	97.58

The next experiment aims to show the significance of the proposed MBWO’s improvement over other swarm intelligence-based hyperparameter optimization algorithms. Table 2 compares the performance of the proposed MBWO with gray wolf optimization (GWO) (Mohakud and Dash 2022), seagull optimization algorithm (SOA) (Aljebreen et al. 2023), equilibrium optimization (EO) (Yang et al. 2023), and whale optimization algorithm (WOA) (Brodzicki et al. 2021) based hyperparameter optimization algorithms. To ensure a fair comparison, all aspects such as the maximum number of iterations, population size, dimension length, searching boundary, and fitness function remain consistent. Other parameters are left unchanged as this paper solely concentrates on enhancing the performance of MBWO. In this table, the mean of the best fitness value and the standard deviation of the best fitness value, as well as the *P*-value of Wilcoxon’s rank sum test with a 5% level, are calculated on the average of independent runs. Wilcoxon’s rank sum test is a non-parametric statistical test. It is chosen for its higher sensitivity than the *t*-test since it assumes proportional differences between matched samples and does not require normal distributions. Furthermore, it is less sensitive to outliers than the *t*-test. A *P*-value of less than 0.05 is generally regarded as sufficient evidence against the null hypothesis. The table demonstrates that the proposed MBWO-based hyperparameter optimization algorithm is very competitive. It achieves the highest mean fitness value coupled with good stability, as evidenced by its minimal standard deviation. Moreover, the obtained *P*-values statistically affirm the remarkable performance of MBWO in comparison to alternative swarm-based hyperparameter optimization.

**Table 2:** The proposed MBWO vs. SOA, GWO, EO, and WOA for hyperparameter optimization in terms of mean, standard deviation, and *P*-value

	Mean	Standard deviation	*P*-value
MBWO	0.838148148	0.01309457	
SOA	0.754814815	0.02618914	< 0.05
GWO	0.791851852	0.07856742	< 0.05
EO	0.791851852	0.05237828	> 0.05
WOA	0.754814815	0.02618914	< 0.05

Table 3 compares the classification results after considering the optimal hyperparameter values obtained from MBWO, SOA, GWO, EO, and WOA. As can be observed from this table, the proposed MBWO-based hyperparameter optimization is a very promising algorithm and can significantly boost the performance of the overall proposed intelligent waste classification model. It obtained the highest accuracy, sensitivity, specificity, and F1-score compared to the other swarm-based hyperparameter optimization algorithms. Additionally, it can be observed that GWO-based hyperparameter optimization is in second place and EO-based hyperparameter optimization. This may be due to that EO has many parameters that need to be adjusted well to get a higher classification result.

**Table 3:** The proposed MBWO vs. SOA, GWO, EO, and WOA for hyperparameter optimization in terms

	Accuracy (%)	Sensitivity (%)	Specificity (%)	F1-score (%)
MBWO	97.57	97.57	99.55	97.58
SOA	96.63	97.75	1	96.66
GWO	97	96.63	99.78	97.03
EO	96.07	95.51	99.78	96.11
WOA	93.07	96.63	99.78	93.26

**Fig. 6:**
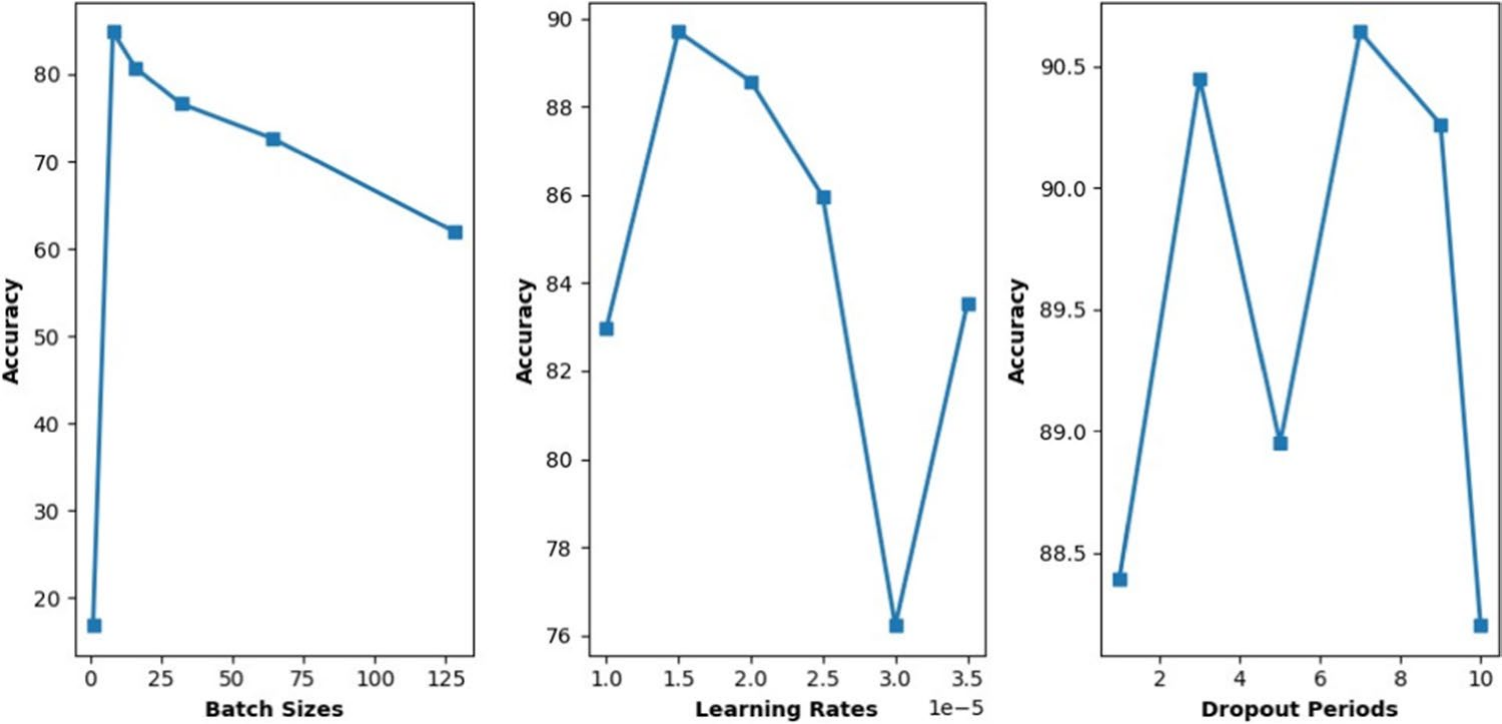
The effect of different values of the dropout period, batch size, and learning rate on the classification accuracy

In order to show how the learning rate, dropout period, and batch size can significantly affect the classification performance of InceptionV3, two other experiments are conducted. Figure 6 compares the obtained classification accuracy for different parameter settings for batch size, learning rate, and dropout period. Finding out which parameter settings had a noticeable impact on the model’s performance was the main objective. In this experiment, the batch size values are in [1, 8, 16, 32, 64, 128], the learning rate values in [0.00001, 0.000015, 0.00002, 0.000025, 0.00003, 0.000035], and the dropout period values in [1, 3, 5, 7, 10] are tested for accuracy outcomes. As can be seen, the batch size is the most significant parameter. Also, when batch size equals 8, the highest classification accuracy is obtained.

In deep learning architectures, the relationship between batch size, learning rate, and model accuracy is complex and influenced by multiple factors (He et al. 2019). Smaller batch sizes often result in better generalization and accuracy due to more frequent parameter updates and overfitting prevention. Larger batch sizes, on the other hand, can improve computing efficiency but may also cause overfitting and slower convergence. The choice of learning rate impacts convergence; too high a rate might cause instability, while too low a rate can result in slow convergence. Changes in one parameter might impact the best option for the other; therefore, the interaction between batch size and learning rate is vital. There were multiple reasons why accuracy decreased, while the batch size was fixed and the learning rate was raised in this experiment. First, divergence during optimization could occur if the learning rate grows too high for the batch size. Secondly, gradient noise can be amplified by higher learning rates when combined with fixed batch sizes, leading to less accurate and less stable updates. Furthermore, optimization dynamics, such as the sensitivity of techniques like stochastic gradient descent to batch size and learning rate, are critical. Overfitting can also result in higher learning rates.

The optimal selection and optimization of different parameters is a vital step in developing and enhancing a deep learning model. The performance, convergence speed, and generalization potential of the model can all be considerably influenced by these parameters. It is crucial to use reliable statistical analysis tools, such as the analysis of variance (ANOVA) and the Kruskal–Wallis test (Usmani et al. 2023). The data are collected for a quantitative dependent variable (classification accuracy) at multiple levels of three independent controlling variables (learning rate, dropout period, and batch size). The accuracy values that we get from running InceptionV3 with different batch sizes, learning rates, and dropout period configurations are what are used to gather the data. The sample size of each equals 6.

**Fig. 7:**
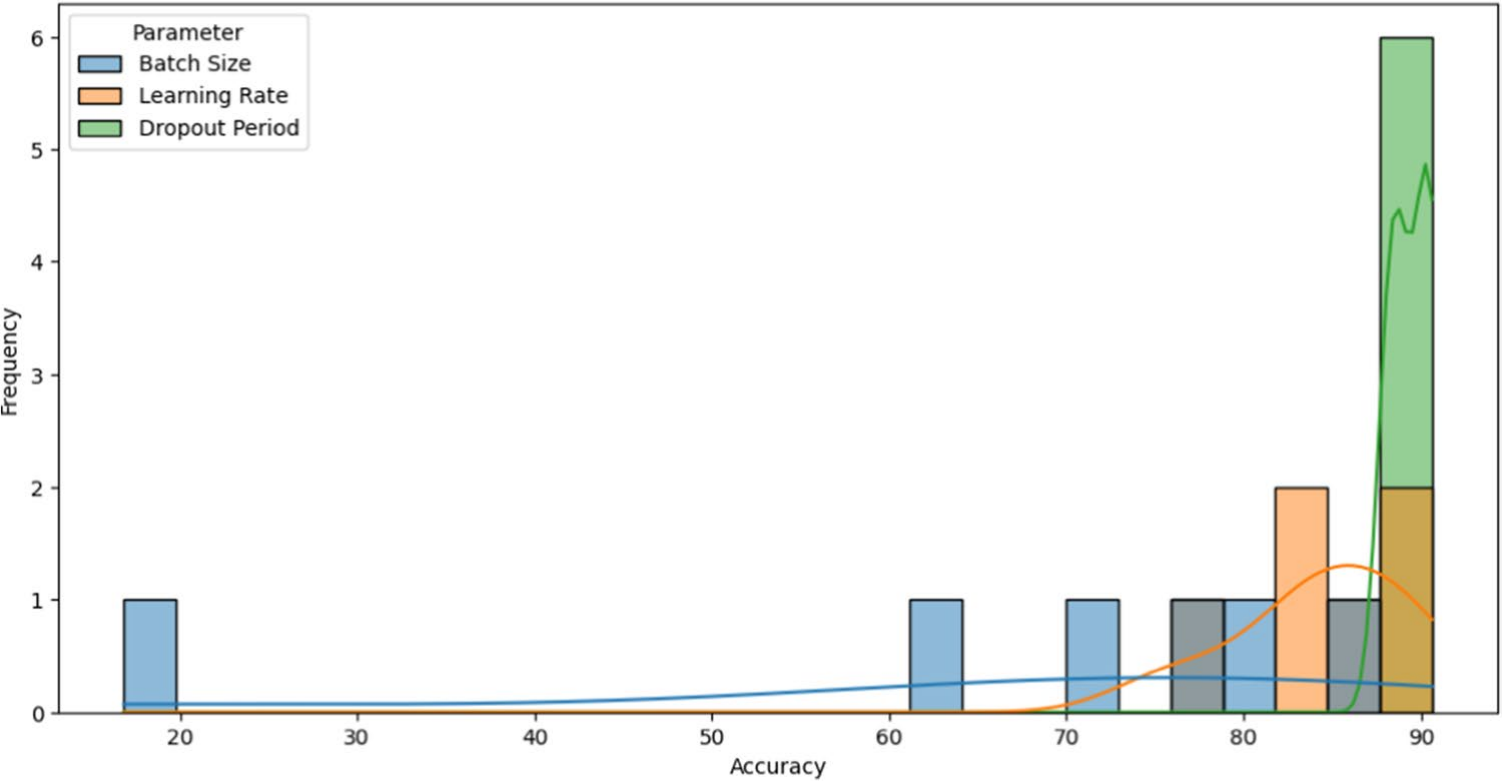
The distribution of accuracy values for different parameter values of the optimized InceptionV3

To assess the suitability of statistical analysis, normality and homogeneity of variance are verified. These tests are common prerequisites for many statistical tests. First, for the normality test, the Shapiro–Wilk test is applied to assess the normality assumption for each variable. A *P*-value less than 0.05 indicates a departure from normality. For the homogeneity test, Levene’s test is applied to examine the homogeneity of the variance assumption. A *P*-value less than 0.05 suggests unequal variances across groups. The *P*-value is the probability of encountering a test statistic as extreme as the one calculated from the sample data, assuming that the null hypothesis is correct. In this paper, the *P*-value of the Shapiro–Wilk test for the batch size equals 0.030971109867095947, while for the dropout period, it equals 0.1664089411497116; and for the learning rate, it equals 0.576654314994812. These obtained values indicate that batch size has a *P*-value < 0.05, indicating a departure from normality. Both the learning rate and the dropout period have a *P*-value > 0.05, suggesting normality. Figure 7 shows the distribution of accuracy values for the dropout period, learning rate, and batch size. As can be observed, the normality of the learning rate is very obvious. These results are consistent to the obtained *P*-values of Shapiro–Wilk. The obtained *P*-value from Levene’s test for homogeneity of variances equals 0.1458279957346559. As can be observed, the *P*-value for Levene’s test is > 0.05, indicating homogeneity of variances across groups.

Given the nature of the non-parametric distribution of the data, the Kruskal–Wallis test is utilized, which does not rely on these assumptions and is more robust in handling non-normally distributed data. In ANOVA, assumptions include normality and homogeneity of variances. Since the batch size violates the assumption of normality, ANOVA cannot be the most appropriate choice. Thus, we considered another statistical test suited for this kind of data in this paper. This statistical test is called the Kruskal–Wallis test. The Kruskal–Wallis test is a non-parametric alternative to ANOVA. Since it does not rely on assumptions of homogeneity of variances or normality and is robust to violations of these assumptions, the Kruskal–Wallis test is the most appropriate statistical test. This decision was made to ensure the validity of our findings and to accurately assess the differences between groups. The H-statistic is the test statistic of the Kruskal–Wallis test. It measures the degree of difference between the medians of the groups being compared. In this paper, the obtained H-statistic equals 11.239766081871352. The obtained *P*-value of the Kruskal–Wallis test is 0.0036250650683908375, which is less than the used significance level of 0.05. The null hypothesis is rejected since the *P*-value is less than 0.05. This indicates that the data is strong enough to conclude that the groups differ statistically. In other words, these findings suggest that the differences in accuracies observed across different batch sizes, learning rates, and dropout periods are unlikely to be due to random chance. Thus, this can indicate the noticeable impact on the performance of the InceptionV3. Therefore, it is important to consider and optimize these hyperparameters to achieve the best possible accuracy for waste classification using InceptionV3.

In a deep learning model, evaluating each parameter separately can be computationally time-consuming and may not always result in the best accuracy, especially when considering parameter combinations. Instead, a swarm algorithm is used to simultaneously search for optimal values across several parameters. The parameter space can be efficiently explored with the adoption of the swarm optimization algorithms, enabling the identification of parameter combination–based synergistic effects. While increasing the possibility of locating the ideal parameter configurations that improve classification accuracy, this approach lowers computational costs. Thus, this paper introduces the MBWO-based hyperparameter optimization algorithm to find the optimal combination of learning rate, dropout period, and batch size that can significantly boost the performance of the InceptionV3 model. The optimal values for the hyperparameters for the InceptionV3 architecture in this paper are a batch size of 32, a drop period of 5, and a learning rate of 0.00015. Significantly, the other parameters have been configured to produce results as rapidly and efficiently as possible. The execution environment is set to multi-GPU, and the number of epochs is restricted to a maximum of 30. Next, the evaluation of the overall performance of the proposed intelligent waste classification model is discussed.

**Fig. 8:**
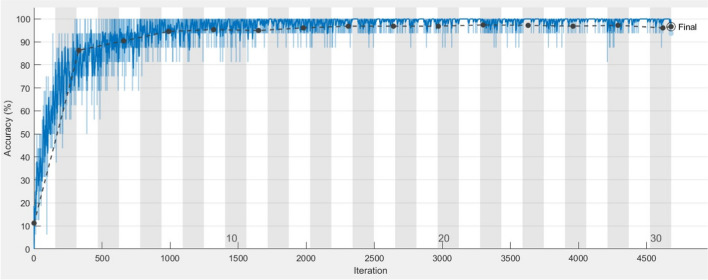
Training progress curve of the optimized InceptionV3

The training progress curve of the proposed intelligent waste classification model, which is based on the proposed MBWO-based hyperparameter tuning algorithm, is shown in Fig. 8. The *y*-axis of the curve represents the evolution of the classification accuracy of the model throughout training. The number of training iterations is represented on the *x*-axis. The proposed MBWO-based hyperparameter algorithm is successful in increasing the model’s accuracy, as shown in the figure. The curve’s form can be employed to spot possible problems as well as provide insights into how well the model is being trained. Overall, the training progress curve shown in Fig. 8 is an effective visualization tool that aids in understanding the effectiveness and optimization of the proposed model.

**Fig. 9:**
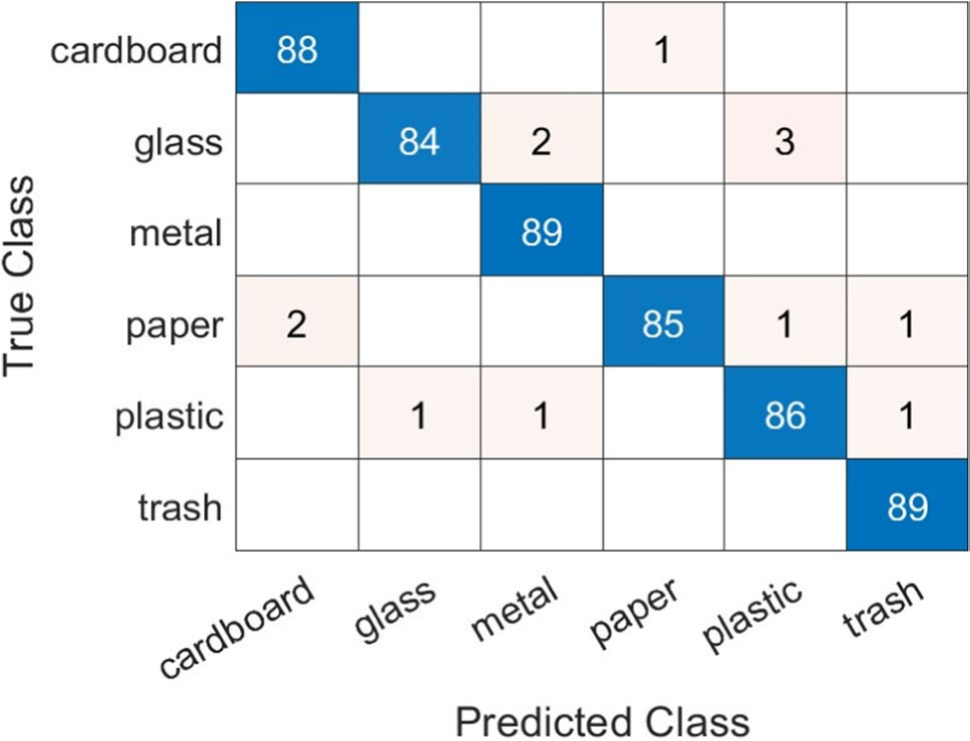
Confusion matrix of the proposed intelligent waste classification model

Figure 9 shows the confusion matrix, which reports the number of true negatives, true positives, false negatives, and false positives, and was considered to assess the performance of the proposed model. The findings of the confusion matrix reveal that the proposed intelligent waste classification model can classify waste materials with high accuracy. The model’s ability to precisely recognize and classify various waste object kinds can be seen by the small number of samples that were not correctly classified across the six groups. Additionally, the results revealed that the proposed model performs exceptionally well at classifying waste by utilizing the InceptionV3 deep learning architecture and hyperparameter optimization with MBWO. Only a small percentage of samples were incorrectly classified, according to the confusion matrix, which shows that the vast majority of samples were correctly identified. Additionally, it can be revealed that the most common errors occur between the “glass” and “paper” classes. This is most likely due to their similar visual characteristics, which pose a challenge to even advanced classification models. To reduce misclassifications, additional features that discriminate between “glass” and “paper” may be included in the model. This could imply more detailed texture analysis or the integration of spectral information. These misclassified samples provide great opportunities for model development. Examining these examples allows us to identify specific features or trends that may be worth paying more attention to. Overall, the model’s performance is commendable, demonstrating its usefulness in waste classification tasks. The low frequency of misclassifications attests to the resilience and accuracy of the proposed intelligent waste classification model in dealing with a wide range of waste materials. Moreover, the results point to the possibility of using the proposed waste classification model in real-world settings to manage waste materials and advance sustainable development.

**Fig. 10:**
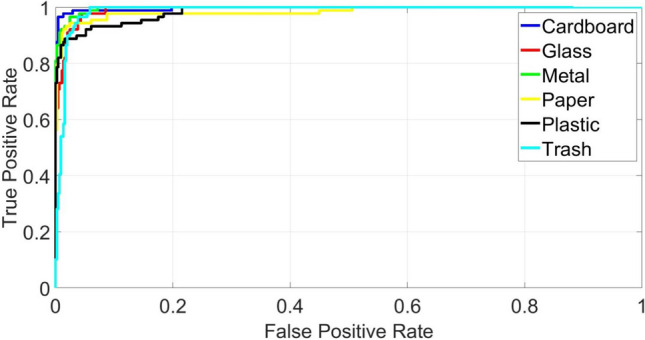
The ROC curve of the proposed intelligent waste classification model

Another experiment is conducted to evaluate the performance of the proposed intelligent waste classification, where the receiver operating characteristic (ROC) curve is utilized. This curve is frequently applied to compare the true positive rate to the false positive rate and assess how well a classification model is working. Figure 10 shows that the proposed model’s accurate classification rate is significantly greater than its misclassification rate. Additionally, the ROC plot’s reported area under the curve is very close to the highest value possible, indicating strong classification performance. These findings imply that the proposed waste classification model can accurately identify and classify different types of electronic waste, making it a potentially useful tool for waste management and environmental sustainability initiatives.

The fourth main experiment in Table 4 aims to compare the proposed waste classification model’s performance utilizing optimized InceptionV3 to three other deep learning architectures in terms of accuracy, F1-score, sensitivity, and specificity. These architectures are Mobilenetv2, AlexNet, and VGG16. According to the results, the proposed intelligent waste classification model based on InceptionV3 performs better than the other deep learning architectures in classifying data. Superior outcomes from the optimized InceptionV3 design showed its potential for use in waste management and environmental sustainability initiatives. The comparative study in Table 4 emphasizes the proposed waste classification model’s potential efficacy and contribution to more effective and efficient waste management procedures.

**Table 4:** Comparative analysis of the performance of the proposed optimized InceptionV3 architecture against other deep learning architectures

	Accuracy (%)	Sensitivity (%)	Specificity (%)	F1-score (%)
MobileNetV2	93.07	97.74	98.65	93.16
AlexNet	92.88	97.75	100	92.91
VGG16	96.44	96.63	99.78	96.57
Optimized InceptionV3	97.57	97.57	99.55	97.58

The last experiment in Table 5 aims to compare the proposed waste classification model’s overall performance against other state-of-the-art models. To guarantee a fair comparison, the models were assessed using the same dataset (the TrashNet benchmark dataset). The outcomes show that the proposed intelligent waste classification model performs better than other state-of-the-art models overall and is a reliable classification model. This shows that the proposed model has the potential to be applied to more complicated datasets and to help develop waste management techniques that are more efficient.

**Table 5:** The proposed intelligent waste classification model performance against the state-of-the-art models

	Algorithm	No. of classes	Year	Accuracy (%)	Sensitivity (%)	F1-score (%)
Yang and Thung (2016)	SVM	6	2016	63.00	58.67	-
Bircanoğlu et al. (2018)	RecycleNet	6	2018	95.00	-	-
Aral et al. (2018)	DenseNet121	6	2018	95.00	-	-
Kennedy (2018)	OscarNet (based on VGG19)	6	2018	88.42	-	-
Adedeji and Wang (2019)	ResNet-50 with SVM	4	2019	87.00	-	-
Ruiz et al. (2019)	Inception-ResNet	6	2019	88.66	-	-
Meng and Chu (2020)	SVM + HOG	6	2020	95.35	-	-
Shi et al. (2021)	MLH-CNN	6	2021	92.6	91.00	91.00
Melinte et al. (2020)	DenseNet121	6	2021	93.33	93.27	92.57
Poudel and Poudyal (2022)	DenseNet201	6	2022	95.05	72.85	72.85
Qin et al. (2022)	Saliency network Salinet with InceptionV3	5	2022	93.24	-	93.06
Abu-Qdais et al. (2023)	JONET deep learning model	6	2023	96.06	-	95.3
Kunwar(2023)	MWaste-based deep learning model	6	2023	92	-	-
Kumsetty et al. (2023)	ResNet-101	6	2023	93.13	-	-
The proposed model	Optimized InceptionV3	6	2024	97.57	95.57	97.58

The proposed waste classification model’s actual implementation, developed and implemented in MATLAB, exhibits a simple integration into real-world waste management processes. To guarantee that the transition from research to practice is as smooth as possible, this system requires only a picture acquired by a common camera, making it an easily accessible and user-friendly solution for waste processing facilities. Adopting the proposed intelligent trash classification model will result in significant long-term cost savings. Facilities can minimize personnel expenses while improving classification accuracy by automating the waste classification process. Furthermore, higher waste processing efficiency may result in increased throughput, increasing economic viability even further. Waste facilities frequently save large image datasets of waste materials. The proposed model is made to easily work with current data gathering and management technologies. Without the need for extensive infrastructure changes, this maintains compatibility with current waste management practices. Because the model was developed in MATLAB, it can be used with a variety of computing resources. Due to its effectiveness, waste classification procedures can be classified in real time, allowing for rapid decision-making. The proposed model can run on typical hardware setups used in waste processing facilities.

## Conclusion and future work

This paper introduces an intelligent waste classification model. The proposed model is composed of two main phases, including data preprocessing and classification based on utilizing the multi-objective beluga whale optimization-based hyperparameter tuning of InceptionV3. The TrashNet benchmark dataset is adopted for evaluating the performance of the proposed model. The experimental results revealed that the proposed optimized InceptionV3 deep learning architecture outperformed MobileNetV2, VGG16, and AlexNet deep learning architectures. Additionally, the results revealed that the random oversampling method and the data augmentation techniques can significantly handle the class imbalance problem of TrashNet and thus boost the performance of the overall waste classification model. Moreover, the results demonstrated that the proposed MBWO can remarkably find the optimal hyperparameter values of InceptionV3 that can significantly boost its classification performance. The results revealed that the proposed MBWO is the optimal hyperparameter optimization of the InceptionV3 algorithm compared to GWO, WOA, EO, and SOA. The proposed intelligent waste classification model obtained an overall accuracy of 97.75%, a specificity of 99.55%, a sensitivity of 99.88%, and an F1-score of 97.58%. In addition, the results showed that, in comparison to the state-of-the-art models, the proposed model does remarkably well at classifying waste images. Moreover, the results demonstrated the potential of the proposed intelligent waste model for use in waste management and environmental sustainability programs in the real world. The results also demonstrated that utilizing the proposed intelligent waste classification model with or without human involvement will speed up and intelligently separate waste. However, there were instances where the proposed model was unable to distinguish between the “glass” and “paper” classes. This is probably because of their comparable visual traits, which even highly developed classification models find difficult to handle. The model may incorporate in the future more features that differentiate between “glass” and “paper” to lower the number of misclassifications. The proposed waste classification model will be further developed in future studies to address more complex waste management issues. Additional exploration into various swarm intelligence algorithms will be pursued through further investigation and experimentation.

## Data Availability

The adopted dataset used during the current study is a benchmark dataset, namely, TrashNet, and can be downloaded from https://www.kaggle.com/datasets/feyzazkefe/trashnet
